# FREQ-Seq^2^: a method for precise high-throughput combinatorial quantification of allele frequencies

**DOI:** 10.1093/g3journal/jkad162

**Published:** 2023-07-26

**Authors:** Roy Zhao, Tamas Lukacsovich, Rebecca Gaut, J J Emerson

**Affiliations:** Center for Complex Biological Systems, University of California, Irvine, CA 92697, USA; Brain Research Institute, University of Zürich, 8057 Zürich, Switzerland; Department of Ecology and Evolutionary Biology, University of California, Irvine, CA 92697, USA; Center for Complex Biological Systems, University of California, Irvine, CA 92697, USA; Department of Ecology and Evolutionary Biology, University of California, Irvine, CA 92697, USA

**Keywords:** genomic methods, genotyping, allele frequency quantification, evolutionary dynamics

## Abstract

The accurate determination of allele frequencies is crucially important across a wide range of problems in genetics, such as developing population genetic models, making inferences from genome-wide association studies, determining genetic risk for diseases, as well as other scientific and medical applications. Furthermore, understanding how allele frequencies change over time in populations is central to ascertaining their evolutionary dynamics. We present a precise, efficient, and economical method (FREQ-Seq^2^) for quantifying the relative frequencies of different alleles at loci of interest in mixed population samples. Through the creative use of paired barcode sequences, we exponentially increased the throughput of the original FREQ-Seq method from 48 to 2,304 samples. FREQ-Seq^2^ can be targeted to specific genomic regions of interest, which are amplified using universal barcoded adapters to generate Illumina sequencing libraries. Our enhanced method, available as a kit along with open-source software for analyzing sequenced libraries, enables the detection and removal of errors that are undetectable in the original FREQ-Seq method as well as other conventional methods for allele frequency quantification. Finally, we validated the performance of our sequencing-based approach with a highly multiplexed set of control samples as well as a competitive evolution experiment in *Escherichia coli* and compare the latter to estimates derived from manual colony counting. Our analyses demonstrate that FREQ-Seq^2^ is flexible, inexpensive, and produces large amounts of data with low error, low noise, and desirable statistical properties. In summary, FREQ-Seq^2^ is a powerful method for quantifying allele frequency that provides a versatile approach for profiling mixed populations.

## Teaser

FREQ-Seq^2^ is a precise, efficient, and economical method for quantifying allele frequencies at loci of interest. FREQ-Seq^2^ can be targeted to specific genomic regions of interest, which are amplified using universal barcoded adapters to generate Illumina sequencing libraries. Available as a kit along with open-source software for analyzing sequenced libraries, it enables detection and removal of errors that are undetectable in the original FREQ-Seq method as well as other conventional methods for allele frequency quantification.

## Introduction

Currently, available sequencing technologies provide vast amounts of data describing genetic variation in a fast and cost-effective manner (Koboldt *et al.* 2013; Park and Kim 2016). Targeting specific alleles with methods that leverage these technologies can produce a wealth of data at a modest cost with substantial sample sizes for the particular genomic regions of interest, which are comparably infeasible using traditional whole-genome sequencing or experimental assays (Kirov *et al.* 2006; Woods *et al.* 2006; Kong *et al.* 2018). Methods for accurately and efficiently quantifying allele frequencies are valuable in a wide variety of biological contexts, such as in tracking candidate genes identified in an association study, constructing and validating population genetic models, and estimating distributions of fitness effects, among other topics (Lynch *et al.* 2014).

The method we report in this study is an extension of a method known as FREQ-Seq (Chubiz *et al.* 2012). FREQ-Seq amplifies loci of interest from mixed population samples using short user-designed oligonucleotides and plasmid-based barcoded bridging primers. The amplification products consist of fragments containing the DNA sequence for a query region of the genome along with a barcoded adapter sequence, where each barcode can be assigned to a specific sample, as well as Illumina sequencing adapters at each end. The resulting libraries can be sequenced to determine allele frequencies in the locus of interest without requiring additional library preparation.

A principal limitation of this method is that every sample within a library requires its own unique barcode, and thus the construction and maintenance of a barcoded adapter plasmid, in order to generate the required bridging primer. Due to the linear scaling of library preparation labor and complexity with the number of samples in an experiment, this can quickly become infeasible for experiments requiring large numbers of samples. For example, the number of samples in data from longitudinal population studies or highly replicated experiments can easily number in the hundreds and thousands (1000 Genomes Project Consortium 2015).

Here, we present a method named FREQ-Seq^2^, in which we apply double-barcoding to achieve a considerable expansion of the method’s throughput and scalability. In particular, our use of two independent barcodes to uniquely label a sample produces a substantial increase in the method’s scalability by allowing the number of samples to scale quadratically rather than linearly, with the complexity of library preparation. At the same time, our method preserves the original advantages of FREQ-Seq, including the ease and flexibility of creating custom libraries for specific experiments. The present method introduces a new plasmid library for preparing sequencing libraries that exponentially increases the number of possible unique labels, with minimal impact on complexity and cost. A FREQ-Seq^2^ library consists of DNA segments spanning the locus of interest, along with two adapter sequences that are tagged with a unique pair of barcode sequences. With 48 unique sequences available for each of the two barcodes, the range of barcoded adapter fragment libraries consists of 2,304 (48^2^) unique combinations that can be used to label and identify samples within a single library.

We demonstrate the real-world performance of FREQ-Seq^2^ on a series of competitive evolution experiments, competing two strains of *Escherichia coli* that differ in an inactivating single-nucleotide polymorphism (SNP) in the L-arabinose isomerase (*araA*) gene over 2,000 generations (Lenski *et al.* 1991; de Visser and Lenski 2002; Tenaillon *et al.* 2012). First, we test several unique combinations of barcodes on a control dataset with known target allele frequencies and quantify the accuracy, precision, efficiency, and throughput that the method achieves. Then, we use FREQ-Seq^2^ to label experimental samples, genotype the samples over the course of a competitive evolution assay and analyze the data to determine changes in allele frequencies and fitness over time. We compare the results of our FREQ-Seq^2^ analysis to estimates obtained from manually counting colonies. Finally, we discuss the combined results of these experiments as well as the utility of FREQ-Seq^2^ for tackling questions in population, evolutionary, and quantitative genetics. Our implementation of FREQ-Seq^2^ includes an available kit with two sets of 48 plasmids containing barcoded adapter fragments as well as fast and efficient open-source software for analyzing sequencing data. Overall, FREQ-Seq^2^ provides a method to measure allele frequencies within and between populations that is accurate, precise, flexible, high-throughput, and economical.

## Methods

### Constructing the barcoded adapter plasmid library

**Fig. 1.**
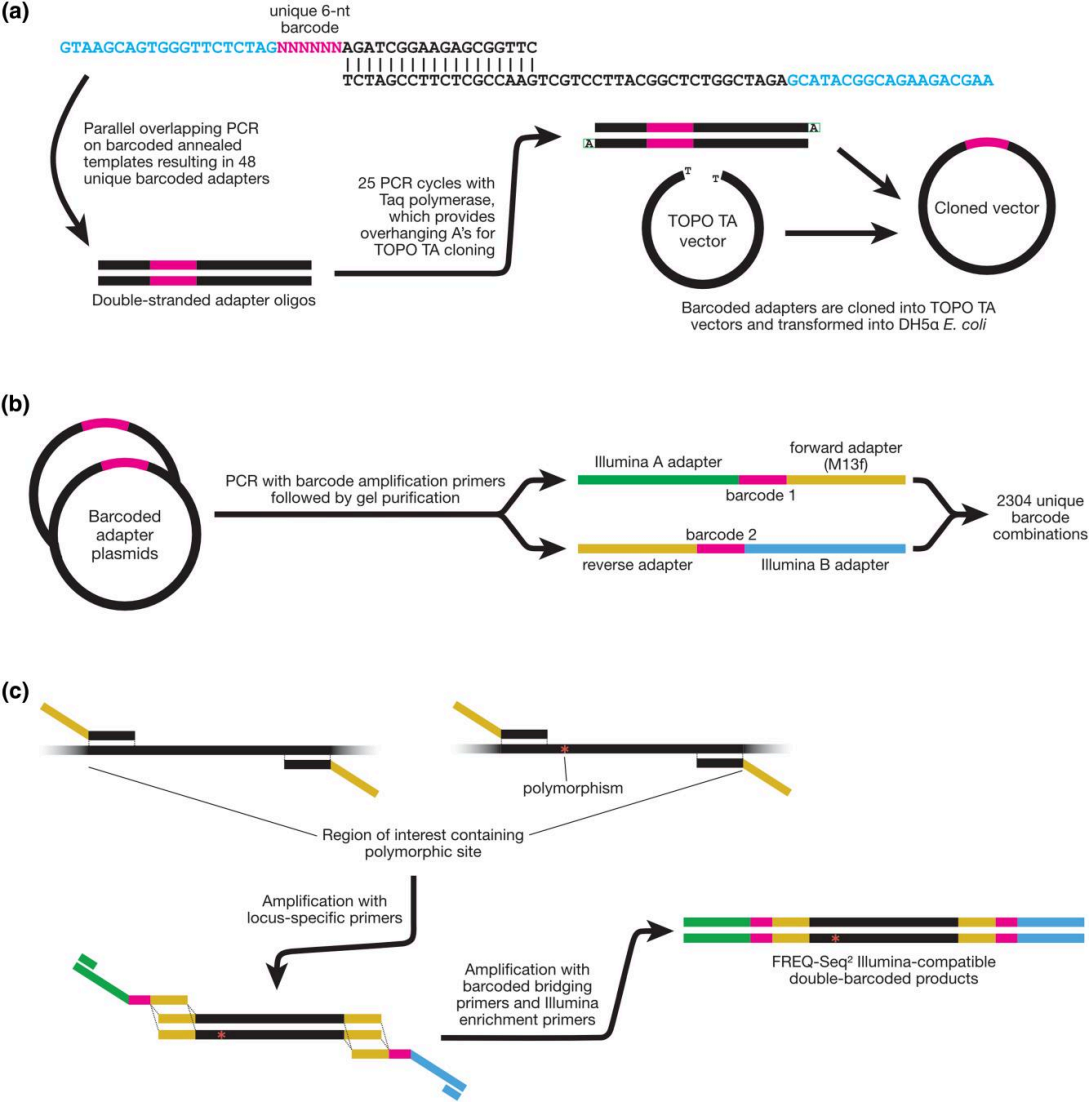
a) Protocol for generating the FREQ-Seq^2^ adapter library. Partially complementary single-stranded oligonucleotides containing the barcodes are annealed together, extended, and PCR amplified with primers corresponding to the regions in blue. Next, they are amplified with Taq polymerase to add overhanging adenosines, for cloning into the TOPO TA vector. After cloning into the plasmids, the vectors are transformed into competent DH5α*E. coli* bacteria and plated, and plasmid DNA is extracted from the transformed bacteria. b) The Illumina-compatible FREQ-Seq^2^ barcoded bridging primers for paired-end sequencing can be amplified from the adapter plasmids using the same amplification primers used to generate the adapter fragments. These adapters can be used in conjunction with their corresponding FREQ-Seq barcoded adapters for double-barcoded labeling of fragment mixtures. c) To generate a FREQ-Seq^2^ sequencing library, amplification is first performed using locus-specific primers to produce a pool of fragments in a region of interest. These fragments contain adapters on each end that are complementary to the barcoded bridging primers, enabling double-barcoded labeling. Amplification is then performed using the barcoded bridging primers and enrichment primers, resulting in Illumina-compatible double-barcoded products.

To enable the double-barcoding in FREQ-Seq^2^, we constructed an adapter library for storing the universal barcoded adapters. The FREQ-Seq^2^ adapter library is stored in a plasmid vector, similarly to that of the original FREQ-Seq method (Chubiz *et al.* 2012). The library utilizes the Thermo Fisher Scientific TOPO TA PCR cloning vector for the plasmid. The 48 double-stranded adapters were generated by 48 parallel overlapping PCR reactions on the annealed template of the partially complementary single-stranded oligonucleotides following the experimental arrangement shown in Fig. 1a, using the forward and reverse amplifying primers AAGCAGAAGACGGCATACG and GTAAGCAGTGGGTTCTCTAG, respectively, analogous to the primers ABC1 and ABC2 from the original FREQ-Seq.

Amplification of the adapter fragments for cloning was carried out with 25 PCR cycles with 15 second elongation periods. The QIAGEN Taq DNA polymerase was used in order to provide overhanging A residues required for TOPO TA cloning. The resulting 87 bp double-stranded oligonucleotides were cloned into the TOPO TA vector following the manufacturer’s recommended protocol for the TOPO TA cloning kit. Half of the reaction mixture (3 μL) from each reaction was transformed into competent *E. coli* DH5α cells provided by the kit. Plasmid DNA was prepared from single white-colored colonies chosen based on blue-white selection and was confirmed via sequence by vector-specific M13f Illumina sequencing primers.

### Generating the FREQ-Seq^2^ sequencing library

With the adapter library available, the barcoded Illumina bridging primers for paired-end sequencing can be PCR amplified from the plasmids of the adapter library, regardless of the orientation of the adapter fragment in the plasmid vector (the TOPO TA cloning of the insert is not orientation-dependent), using the same small forward and reverse amplifying primers that were used for the parallel overlapping PCR to generate the adapter fragments. These amplified adapters were gel-purified on 2% agarose gel and then used in conjunction with their corresponding original FREQ-Seq barcoded adapters for double-barcoded labeling of the fragment mixtures.

Amplification of the specific region of interest is performed using the following primers, complementary to ABC2 from the original FREQ-Seq and the FREQ-Seq^2^ reverse amplifying primer described above: GTAAAACGACGGCCAGT plus a 20-nucleotide locus-specific forward primer, and CTAGAGAACCCACTGCTTAC plus a 20-nucleotide locus-specific reverse primer. The PCR reaction was carried out using the Thermo Fisher Phusion DNA polymerase, and the resulting PCR products were gel-purified on 2% agarose gel to remove unincorporated primers and diluted 100-fold for the second stage of PCR.

For barcoding of the amplified mixtures, the diluted templates were PCR amplified with Phusion DNA polymerase using 10X molar equivalent of primers ABC1 from the original FREQ-Seq and the FREQ-Seq^2^ forward amplifying primer (0.1–0.2 μM) against the original FREQ-Seq and the FREQ-Seq^2^ purified adapters (10–20 ng). The pooled barcoded amplification products, consisting of a proportional mixture of the sequences from different samples, constitute an Illumina-compatible library for paired-end sequencing. A final purification step (e.g. using a gel or Pippen) may be performed at this stage if desired to remove residual adapters and primers.

### Estimating fitness of evolved strains

To examine the application of FREQ-Seq^2^ in a real-world evolutionary biology application, we performed competition assays in which an evolved strain of *E. coli* was competed against an ancestral strain to estimate the adaptive trajectory of the evolved strain’s relative fitness. At several time points over 2,000 generations, the evolved strain was competed against the ancestral strain, and their relative frequencies were measured and used to establish a fitness trajectory for the evolved line.

The evolved strains had previously been serially propagated for 2,000 generations in Davis minimal broth supplemented with 25 mg/L of glucose (DM25) at 42.2${}^{\circ }$ and periodically stored as frozen glycerol stocks at $-80^{\circ }$ (Carlton and Brown 1981; Tenaillon *et al.* 2012). These strains originated from a clone of *E. coli* B strain REL1206, which was isolated from the *E. coli* long-term evolution experiment (LTEE) and possesses an *Ara*${}^{-}$ neutral marker (Lenski *et al.* 1991). REL1206 had been evolved for 2,000 generations at 37${}^{\circ }$ in the LTEE and so was adapted to the DM25 medium. The ancestral strain used for the competitions, REL1207, is equivalent to REL1206 aside from possessing a single-nucleotide *Ara*${}^{+}$ mutation.

For each generation, a sample of the evolved strain and of the ancestral strain were each collected on a sterile loop from frozen glycerol stock, inoculated into 10 mL of Luria-Bertani (LB) broth, and incubated at 37${}^{\circ }$ overnight in a shaking water bath. For each strain, the culture was diluted 100-fold in phosphate-buffered saline, and 0.1 mL was transferred into 9.9 mL of DM25 and incubated at 37${}^{\circ }$ for 24 h. Then, 0.1 mL of each culture was transferred into 9.9 mL of DM25 and incubated at 42.2${}^{\circ }$ for 24 h. From their respective incubated cultures, an aliquot of the evolved strain (*Ara*${}^{-}$) along with an aliquot of the ancestral strain (*Ara*${}^{+}$) were transferred to a 1.5 mL centrifuge tube, and the tube was vortexed. For the colony counting samples, 0.025 and 0.225 mL were transferred of the evolved and ancestral lines, respectively, over six replicates. This protocol was repeated for the FREQ-Seq^2^ samples, with 0.005 and 0.245 mL transferred of the evolved and ancestral lines, respectively, over eight replicates to optimize the utilization of a 96-well plate. The ratios of the strains in the centrifuge tubes represent the initial (prior to competition) frequencies.

We used small initial proportions of *Ara*${}^{-}$ in order to increase the resolution and decrease the measurement error in the downstream fitness calculations since these strains have substantially different fitness from the ancestral strain due to the adaptive environment under which the *Ara*${}^{-}$ strain was previously propagated. In a competition assay, as the gap in the relative fitness between competing strains increases, the measurement error increases when the counts of the lower-fitness ancestor (in the denominator in the fitness calculation) become increasingly small and difficult to quantify (Wiser and Lenski 2015). The precision and sensitivity of FREQ-Seq^2^ enabled the use of a very small initial frequency (2%) of the *Ara*${}^{-}$ strain. A target 10% *Ara*${}^{-}$ initial frequency was used for colony counting, as the 2% initial frequency for visual measurement was not feasible due to insufficient visual signal for the pre-competition counts.

The pre-competition mixtures were created by transferring 0.1 mL from each centrifuge tube to a culture tube containing 9.9 mL of DM25. For the colony counting samples, 0.1 mL of a 100-fold dilution from each pre-competition mixture was plated on tetrazolium arabinose (TA) agar to obtain measurements of the initial frequencies. The culture tubes were incubated at 42.2${}^{\circ }$ for 24 hours to compete the strains. For the colony counting samples, 0.05 mL of a 10,000-fold dilution from each post-competition mixture was plated on TA agar. When plated on TA agar, *Ara*${}^{-}$ and *Ara*${}^{+}$ colonies appear red and white, respectively. A visual measurement of the distribution of the evolved strain versus the ancestral strain was taken by counting the plated colonies. For the FREQ-Seq^2^ samples, genomic DNA from each pre-competition and post-competition mixture was extracted using the Promega Wizard Genomic DNA Purification Kit. The FREQ-Seq^2^ sequencing library was prepared as described above with the locus-specific forward primer containing a 20-nucleotide flanking sequence upstream of the allele of interest and a unique combination of barcoded adapters for each sample. Following library preparation, the samples were paired-end sequenced on an Illumina HiSeq 2500 system.

### Obtaining allele frequencies from barcoded reads

Sequencing data from a FREQ-Seq^2^ library can be directly processed by our open-source software tool, *fsdm*, from the raw FASTQ files. The sequencing reads are analyzed to compare each read to the library’s barcode, adapter, and allele sequences in order to identify which samples the reads belong to. These sequences can be specified by the user and are provided to the program in a FASTA file.

Reads are demultiplexed first by matching the sequence of segments at the beginning and end of each read to the barcode pairs used in the library preparation utilizing a hash table optimized for this application. The barcode pair with which each read is labeled is identified, filtering out reads that are not valid FREQ-Seq^2^ reads if they lack a valid barcode combination according to the predetermined sequence information. Then, the adapter sequences and the regions flanking the query allele are extracted from the reads and their sequences are compared against the user-specified sequences. Each read is either verified as a match, up to a user-specified threshold of mismatches, or it is filtered out as an invalid read. Last, reads with an allele matching one of the possible genotypes are recorded, and reads containing unrecognized sequence for the allele are filtered out.

After filtering out reads with unmatching barcode, adapter, flanking, and target allele sequences, the counts for each allele are quantified. The relative frequencies of each allele within a given sample are obtained by dividing the read count for each allele by the total number of valid reads matching the sample’s barcode pair. The software reports the computed frequency for all 2,304 combinations of FREQ-Seq^2^ barcodes. In the results presented here, no mismatches were allowed in the barcodes, and a maximum edit distance of four was allowed across the adapter and flanking sequences for a read pair.

### Demultiplexing and read rescue algorithms

Barcode sequences are identified by comparing the corresponding regions within each read pair to the set of possible barcode combinations using a fast hash table lookup. Barcode comparisons are performed for exact sequence matches as well as an optionally specified single-nucleotide mismatch threshold based on Hamming distance. In the case of allowed mismatches in barcodes, reads are only assigned to a particular barcode combination if the mismatching sequence is not ambiguous, that is, the sequence is not within the same Hamming distance to two or more possible barcodes (Hamming 1950).

Mismatches in the adapter and flanking regions of each read are determined using the Damerau–Levenshtein distance, an edit distance metric which accounts for substitutions, insertions, deletions, and adjacent transpositions (Damerau 1964; Levenshtein 1966). The Damerau–Levenshtein distance is computed between each of the specified adapter and allele flanking sequences and the corresponding portions of the read pair. Reads that exceed the specified edit distance threshold in the adapter and flanking sequences are discarded.

For reads that uniquely match a barcode pair and match within the edit distance threshold for the adapters and flanking sequences but fail to match to a recognized allele, a rescue algorithm is employed to find and genotype reads which contain shifted sequences due to a small insertion or deletion. The reference flanking sequence to the left of the allele (in $5^{\prime }$ to $3^{\prime }$ orientation) is aligned to the corresponding region in the read using a Needleman-Wunsch optimal global sequence alignment (Needleman and Wunsch 1970). If the alignment contains an overhang, indicating the presence of a small indel in the read, the shift is corrected by reindexing the read according to the length of the overhang. The allele position of the read is once again queried and recorded if it matches a recognized allele sequence.

### Calculating relative fitness

We calculated the relative fitness of the evolved *Ara*${}^{-}$ strains as $w_{E}=1+s$, where *s* is the selection coefficient:


(1) $$\log\;(1+s)=\frac{\log\;\frac{\;f_{E,t}/\;f_{A,t}}{\;f_{E,0}/\;f_{A,0}}}{T}.$$

*f* is allele frequency, subscripts *E* and *A* represent the evolved and ancestral strains, subscripts 0 and *t* represent the initial time point and the time at which fitness is estimated, and *T* is the number of generations (Travisano and Lenski 1996; Gillespie 2004). *T* was calculated as $\log_{2}\;100$ based on a 100-fold dilution from stationary phase at the start of each competition assay.

### Power law model

To verify that the difference in the magnitude of *Ara*${}^{-}$ allele frequencies derived from colony counting compared to the FREQ-Seq^2^ data is a result of the higher initial frequencies in the colony count samples, we compared the *Ara*${}^{-}$ frequencies measured after competition at each time point to those under a power law model of fitness increase under asexual adaptation in a constant environment.

Previous research on data obtained from the *E. coli* LTEE has demonstrated that the trajectory of relative fitness increase is well described by an offset power law relating mean fitness as a function of time in generations. The power law fitted to a subset of the data from a set of LTEE populations accurately predicts later measurements, and the addition of clonal interference and diminishing-returns epistatis to a population dynamics model of mean fitness produces power law dynamics (Wiser *et al.* 2013). This power law relationship can be expressed in the form of


(2) $$\bar{w}=(at+1{)}^{b},$$

where $\bar{w}$ represents mean relative fitness and *t* represents time in generations, with two model parameters *a* and *b*.

We fitted the above model to the fitness trajectory for the FREQ-Seq^2^ samples with non-linear least squares regression using the Levenberg–Marquardt algorithm. This power law model allows us to estimate the expected post-competition frequencies for the colony count samples, conditioned on their initial frequencies and with a model derived from an independent dataset obtained via an independent method with separate initial conditions.

We used the fitted model to obtain model predictions of mean fitness for the *Ara*${}^{-}$ strain at each time point. Using these fitness predictions and the measured initial frequencies for the colony counts, we solved for the expected post-competition frequencies in accordance with Eq. (1) at each time point:


(3) $${\hat{\bar{w}}}_{E}={\left(\frac{\frac{{\bar{\;f}}_{E,t}}{1-{\bar{\;f}}_{E,t}}}{\frac{{\bar{\;f}}_{E,0}}{1-{\bar{\;f}}_{E,0}}}\right)}^{1/T},$$

where ${\hat{\bar{w}}}_{E}$ represents the model predictions of mean relative fitness of the evolved strain.

### Statistical analyses

Competitions between the *Ara*${}^{+}$ and *Ara*${}^{-}$ strains were performed independently, and were measured using both the FREQ-Seq^2^ and colony counting methods. We used an analysis of variance (ANOVA) to test for a correlation between the error in estimated allele frequency and barcode. We additionally used a two-way ANOVA to check for the existence of interactions between the method of determining frequency and the replicates at each time point. A significance level of 0.05 was used. Confidence intervals were estimated using a nonparametric empirical CDF-based method, which does not assume that the data follows a particular distribution, and the standard error of the mean.

## Results

### Accuracy and precision

**Fig. 2.**
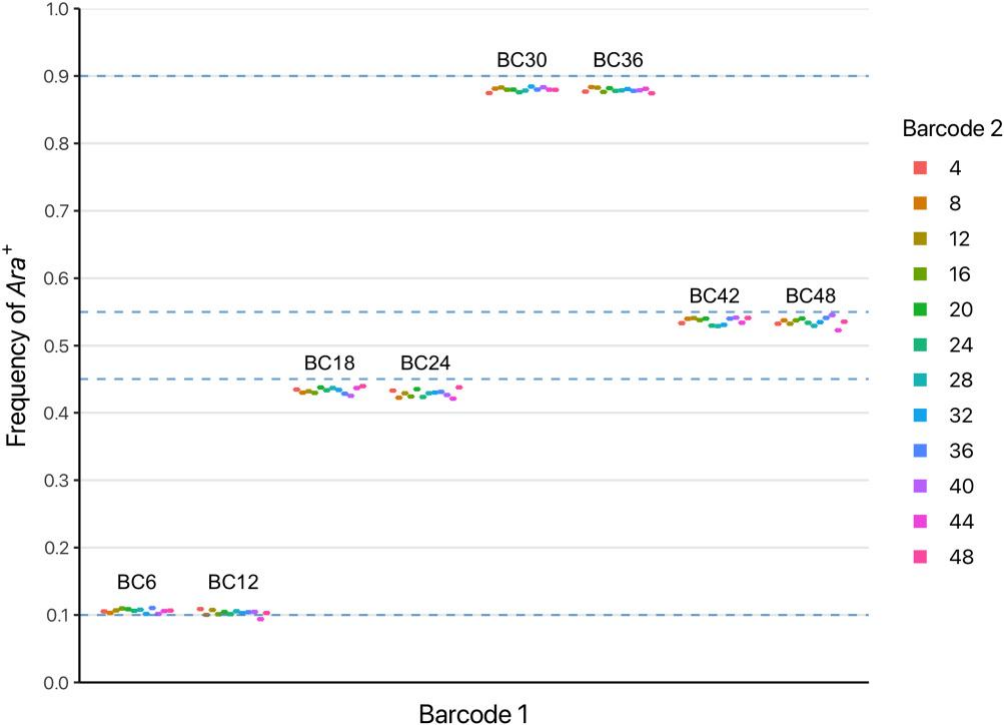
Estimated *Ara*${}^{+}$ allele frequencies using FREQ-Seq^2^ for 96 independent loading controls with unique barcode combinations. Dashed blue lines represent the four target allele frequencies of *Ara*${}^{+}$ that were used to benchmark the controls.

To test the accuracy of FREQ-Seq^2^, we generated libraries from control samples with known relative DNA concentrations and compared the allele frequency estimates obtained with FREQ-Seq^2^ to the target values for each sample. Our test dataset is comprised of 96 control samples consisting of combinations of 8 separate barcodes for the first adapter with each of 12 barcodes for the second adapter. Additionally, we tested four different frequencies of *Ara*${}^{+}$ set at 0.1, 0.45, 0.55, and 0.9. The estimated allele frequencies for all the control samples compared to their target values are shown in Fig. 2.

Variance in FREQ-Seq^2^ allele frequency estimates is small and tightly clustered near the target frequency for a broad range of values. The average error in allele frequencies estimated using FREQ-Seq^2^ in the control samples was 1.47%, with a standard deviation of 0.73%. Note that this estimate of error accounts for not just the variance in the method itself, but also external sources of error, such as sequencing error, contamination, and pipetting error introduced in creating the test samples. Error statistics for the control samples are summarized in Table 1. To investigate whether the method exhibits biases, we examined the distribution of errors and looked for the existence of correlated errors, as deficiencies in these metrics can indicate systematic bias in PCR amplification or sequencing (Acinas *et al.* 2005; Ross *et al.* 2013). The error was not correlated with the barcode sequences at either of the possible positions and is close to normally distributed (Supplementary Fig. 2).

**Table 1.** Error in control sample allele frequency estimates.

Error	Percent
Average error	1.47%
Minimum error	0.02%
Maximum error	2.91%
Standard deviation	0.73%
95% confidence interval	(0.12%, 2.72%)^*a*^

Nonparametric confidence interval based on the empirical cumulative distribution function of the observed errors in allele frequency estimates.

### Testing FREQ-Seq^2^ on real biological samples

To evaluate the performance of FREQ-Seq^2^ with real biological samples, we used the method to obtain allele frequency estimates over evolutionary time for a competition experiment between two strains of *E. coli* that differ at a SNP in the *araA* gene. These estimates were then used to compute the fitness trajectory of this experiment. The *araA* gene encodes the L-arabinose isomerase protein, and is part of the L-arabinose operon. One of the strains we use (*Ara*${}^{-}$) possesses an inactivating SNP in the gene (Cleary and Englesberg 1974), and is routinely used as a neutral visible marker in experimental evolution studies (Lenski *et al.* 1991). Two independent competition assays were performed, in which several independent aliquots of the *Ara*${}^{+}$ strain and evolving *Ara*${}^{-}$ strain were taken and amplified together at each of eleven evolutionary time points spaced over the course of 2,000 generations.

**Fig. 3.**
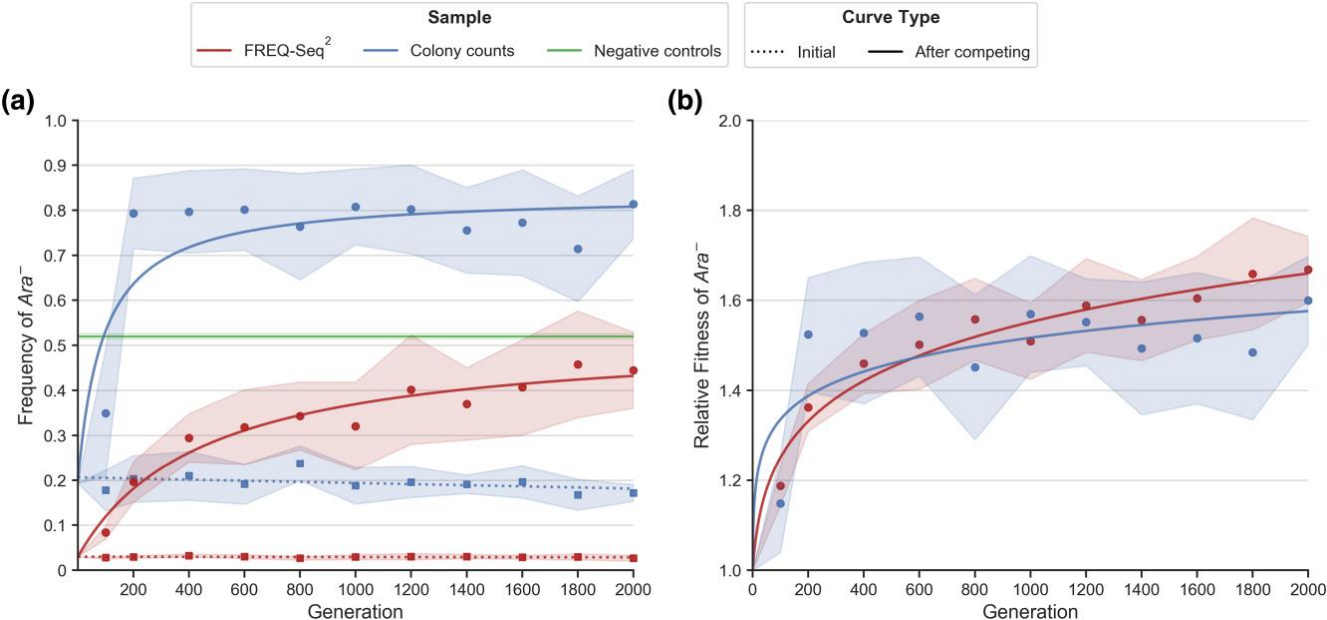
FREQ-Seq^2^ allele frequency and fitness trajectories over time for the evolved *Ara*${}^{-}$ strain. The *Ara*${}^{-}$ strain competed with the ancestral *Ara*${}^{+}$ strain, and their frequencies were measured at several time points over 2,000 generations. a) *Ara*${}^{-}$ allele frequency and b) relative fitness across eleven generations of the competition assay measured using both FREQ-Seq^2^ and manual colony counting. The blue and red dots represent the mean allele frequency or relative fitness at each time point. In a), the dotted lines correspond to the initial *Ara*${}^{-}$ frequency before the strains were conducted and the solid lines correspond to the *Ara*${}^{-}$ frequency after competing. The line and curves show the fit of a linear, hyperbolic, and power law model to the initial frequencies, post-competition frequencies, and fitnesses, respectively. Note that the higher magnitude of the *Ara*${}^{-}$ frequencies for colony counting are due to the higher initial frequencies. The green line is the mean allele frequency measured using FREQ-Seq^2^ for sixteen independent target 50/50 negative controls. The shaded regions represent 95% confidence intervals based on the standard error of the mean.

We used FREQ-Seq^2^ to determine the allele frequency for both of the strains at each time point and then estimated relative fitness based on the allele frequency estimates using the method described by Lenski *et al.* (1991). The frequency and fitness trajectories for the competitions are shown in Fig. 3a and b, respectively. The *Ara*${}^{-}$ allele frequency and relative fitness both increase steadily over the 2,000-generation experiment and on average exhibit near-monotonic upward trajectories. Notably, with a comparatively small number of samples and generations, the characteristics of the FREQ-Seq^2^ frequency and fitness trajectories in our *E. coli* competition assay resemble those of the extensive *E. coli* long-term evolution experiment (Lenski and Travisano 1994; Wiser *et al.* 2013).

The observed variation in fitness trajectories among the different samples at each time point is not necessarily surprising. First, noise inherent in the various steps of a competition assay produces some degree of variation between samples. Second, stochasticity in the traversal of rugged evolutionary fitness landscapes naturally causes rises and dips in fitness on the path towards an optimum (Schoustra *et al.* 2009). This principle regarding evolutionary trajectories with respect to fitness landscapes, including individual sample variation in frequency and fitness at each time point measured in our experiment, has been observed in a wide range of experiments (Collins *et al.* 2007; Schoustra *et al.* 2009; Heredia *et al.* 2017). Additionally, the mean fitness measured in our samples exhibits an initial increase within the first few hundred generations, followed by an eventual deceleration in the fitness increase over time, which is consistent with theoretical expectations as well as the results of long-term studies in experimental evolution (Fisher 1930; de Visser and Lenski 2002; Orr 2009).

### Comparing FREQ-Seq^2^ to manual quantification methods

We compared the estimates of allele frequencies and fitness determined using FREQ-Seq^2^ to those computed by manual colony count measurements. Plating and competitions were performed at the same 11 time points that were used for the sequenced data. Colony counts of each allele were obtained at each generation for all samples and replicates. The mean allele frequency trajectories of *Ara*${}^{-}$ determined by the FREQ-Seq^2^ and colony counting methods are shown alongside each other in Fig. 3a.

Compared to the estimates of allele frequency and fitness determined by colony counts, the FREQ-Seq^2^ data produced more stable measurements for both frequency and fitness, as well as trajectories that more closely match predictions from theory for a population adapting to a fixed environment over time (Fisher 1930; Crow 2002). This was particularly true for relative fitness, where the estimates derived from manual counting exhibited much less stable measurements over time along with substantially higher variance (Fig. 3). The FREQ-Seq^2^ fitness measurements produce a trajectory that exhibits a gradual reduction in the average rate of fitness increase over time characteristic of classic adaptive walks, following an initial increase before generation 400 (Orr 2009; Heredia *et al.* 2017).

The substantially higher magnitude of the post-competition allele frequencies for the colony counts versus FREQ-Seq^2^ is a predictable consequence of the initial *Ara*${}^{-}$frequency in each of the experiments (Fig. 3a). We confirmed this is the case by considering that the adaptive dynamics of fitness in clonal populations is consistent with a power law relationship of mean fitness as a function of time in generations (Eq. 2) (Wiser *et al.* 2013). Fitting this power law to the fitness trajectory derived from the FREQ-Seq^2^ data, computing the relative fitness predicted at each time point using the fitted model, and then solving for the expected post-competition allele frequencies in the colony count samples given their initial frequencies shows that the higher magnitude in the observed post-competition frequencies tracks with expectations (Supplementary Fig. 3).

The larger variance and greater degree of jaggedness in the colony count-based allele frequency and fitness estimates illustrate a major practical benefit of FREQ-Seq^2^’s accuracy and precision, particularly with smaller numbers of samples and degrees of replication. Though this may be mitigated to a degree with larger datasets and increased replication, such changes entail additional costs and labor, or may not be readily available depending on the difficulty in obtaining and preparing samples. As a quality control measure, a negative control sample targeting a 50/50 distribution of *Ara*${}^{+}$ and *Ara*${}^{-}$ was included with each group of FREQ-Seq^2^ samples from our competition assay during library preparation, with sixteen independent negative controls in total. The frequency measurements for the initial frequency at each time point in combination with the negative controls demonstrate that substantial variations between different samples or time points are unlikely to be an artifact of the FREQ-Seq^2^ method itself. Both the negative controls and the initial frequencies are extremely consistent, falling within a very narrow range of variation and closely tracking the target aliquot ratio across all time points. No statistical interaction was observed between the different replicates at each time point and the method used to measure allele frequency.

### Coverage, noise, and resolution

We used the control samples to evaluate the random variation of our method. We compared our frequency observations in these controls to the frequencies among barcode combinations that were not introduced into the experiment. These combinations represent a class of false positives against which we measure the intended barcode combinations. The false positive barcode combinations are divided into two categories. The first is for combinations matching two possible combinations of barcodes that actually exist in the library. The second represents the case where either barcode matches a barcode actually in the library but not both. Notably, the single spurious match category is an exaggerated estimate of the degree of barcode hopping given that contaminating a real category would require matching both barcodes. Nevertheless, we present these results as a conservative upper bound demonstrating how rare errors are. Thus, counts in the first category correspond to an upper bound for the risk of misidentifying a particular sample based on a specious barcode pair, derived from fragments of one or more samples that were erroneously barcoded at any point prior to sequencing (Tanabe and Ishida 2017) (see *Discussion* for additional explanation).

When the frequency of these spurious barcodes approaches that of the lower coverage control samples among the expected barcode combinations, the risk of undetected error increases for allele frequency estimates in these lower coverage samples. Comparison of the three categories (two spurious barcode combinations and the true barcode combinations expected from control groups) shows that the potential for contamination via barcode misassignment is quite low, with the distribution of the single spurious barcode category not overlapping that of the true category. The two spurious distributions share a substantial degree of overlap, and neither class of errors represents an appreciable risk of confounding (Fig. 5a).

**Fig. 4.**
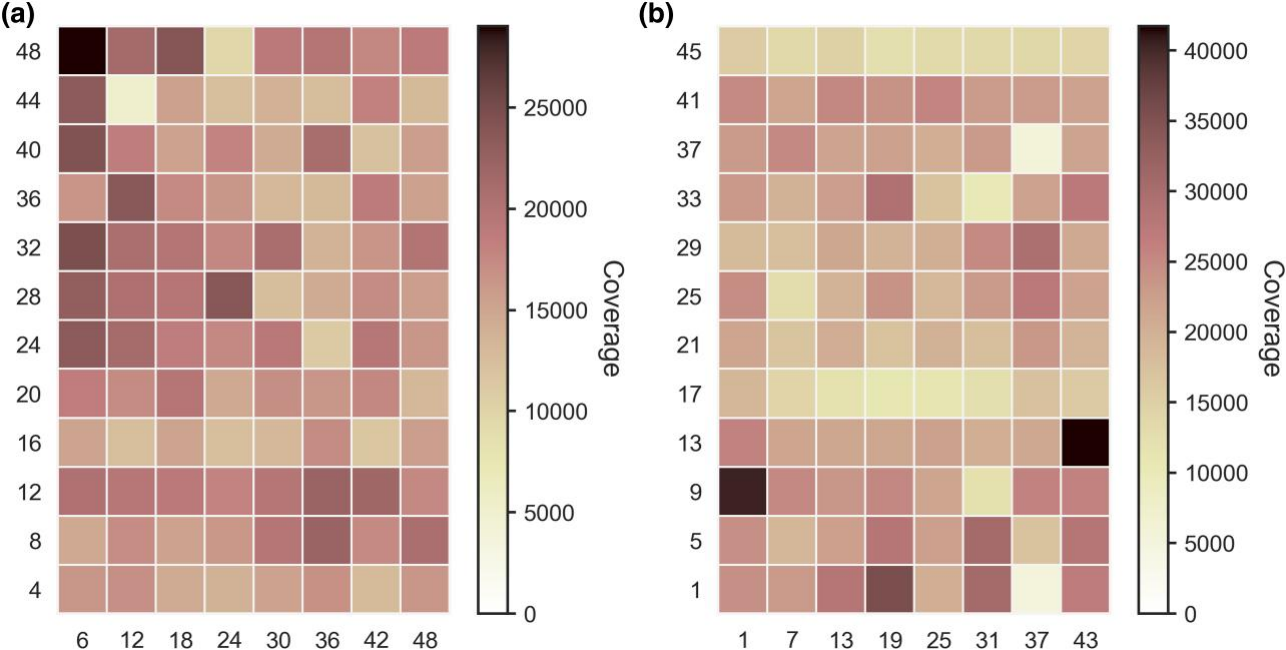
Sequencing read coverage measured for the FREQ-Seq^2^ barcode combinations used in the control and experimental samples. Different sets of 96 distinct barcode pairs were used to label the loading controls and experimental evolution samples, which are clearly identifiable by coverage from the background noise. The labels on the *x*-axis and *y*-axis show the first and second barcodes used to label each of the 96 sample barcode pairs in each heatmap for a) control samples and b) experimental evolution samples. Coverage for barcodes outside the barcode combinations used for sample labeling represents spurious signal from noise in the method or errors during preparation and sequencing.

**Fig. 5.**
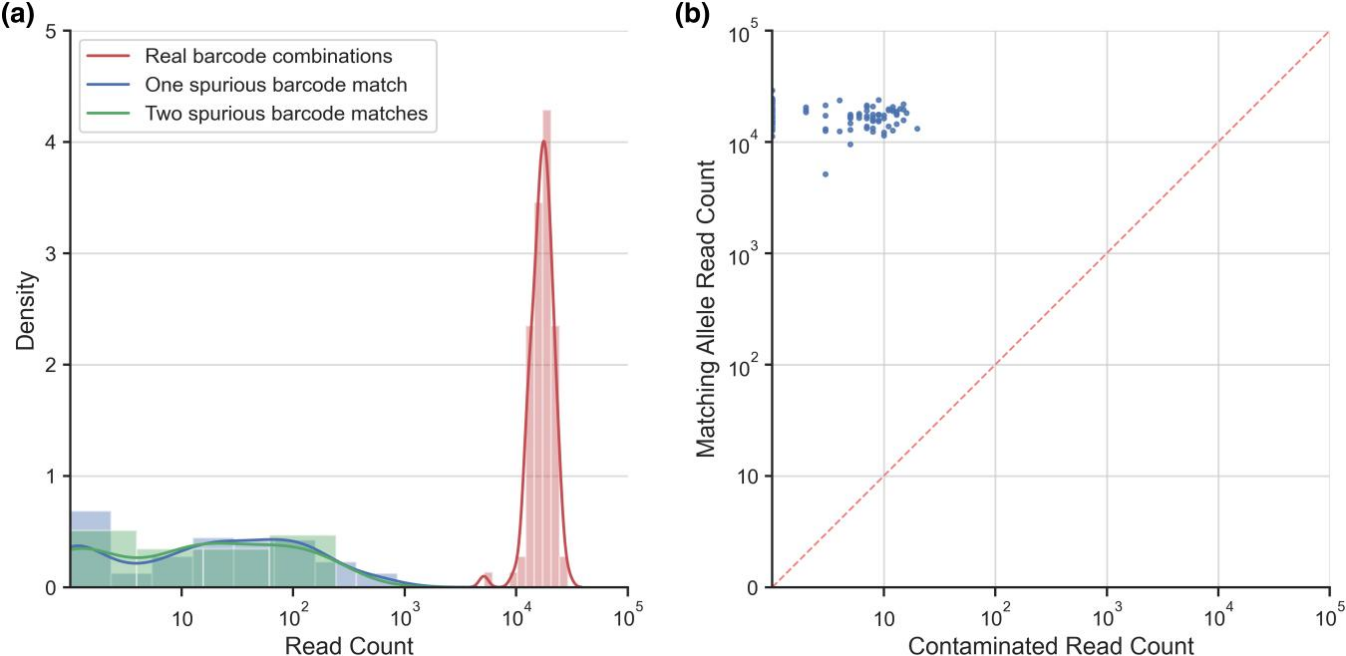
a) Histograms comparing the coverage of properly barcoded reads to that of reads with either one or two improper barcodes for 96 unique control sample barcode combinations. The distributions of one and two spurious barcode matches represent the relative risk of misbarcoding in a FREQ-Seq^2^ library. b) Coverage of reads containing a valid genotype (*y*-axis) versus the coverage of contaminated reads containing an unrecognized allele (*x*-axis) among properly barcoded control sample reads for each of the 96 barcode combinations. The dashed red line is a one-to-one scaled diagonal between the axes.

The coverage for the 96 FREQ-Seq^2^ barcode pairs in our control and experimental samples (a total of 192 unique combinations) are visualized in Fig. 4. Different samples in our library obtained a range of coverage levels, though we did not observe any particular barcode being associated with unusually low or high efficiency. Additionally, the lowest-coverage sample in our library, which was sequenced using a small fraction of a single lane, still produced a read count in the thousands, with the highest sample size reaching well over 10,000. The vast majority of FREQ-Seq^2^ reads are uniquely identified as one of the true combinations in the sequencing library. The coverage of the expected barcode combinations was substantially higher than that of any spurious combinations when comparing across all 2,304 possible barcode pairings. In fact, the coverage of erroneous barcode combinations only approaches within an order of magnitude of the coverage of properly barcoded reads at the very bottom of the coverage distribution (Fig. 5a).

### Throughput, efficiency, and scalability

To evaluate the throughput and scalability of the FREQ-Seq^2^ method, we first looked at the distribution of reads that contain a matching barcode pair but do not contain a proper allele. This statistic examines the accuracy of the barcoding protocol itself, and therefore, the likelihood of correctly identifying a particular sample based on FREQ-Seq^2^ reads. Figure 5b shows the coverage ratio of reads containing a proper allele to those with a mismatched allele for the set of reads with a matching barcode pair. This represents a desirable result, as the number of erroneous reads is far lower than the number of reads with a proper allele for all 96 barcode combinations. Additionally, the distribution does not show a correlation between barcode and error rate.

Next, we examined the frequency distribution of sequencing reads generated from our libraries. Specifically, we investigated the rank-frequency distribution of reads that contain both a valid combination of FREQ-Seq^2^ barcodes and a matching target allele sequence, representing the true positives. This statistic is useful for evaluating the method’s effective throughput relative to total coverage, as it looks specifically at the reads that are usable for downstream analyses. To gauge the representation of usable reads compared to erroneous reads, we computed the worst-case coverage ratio of the true positive samples to the highest-coverage erroneous barcode combination comprised of two individually valid barcodes. The rank-frequency distribution for our control samples is shown in Supplementary Fig. 1. Mirroring the results in Fig. 4, the rank-frequency distribution indicates that the method produces comparable and substantial coverage for the vast majority of samples in a library, and thus will scale well by simply increasing the library size until a desired sample size is reached.

Finally, to evaluate the overall efficiency of the FREQ-Seq^2^ pipeline, we examined the overall rate of useful reads generated from our barcoded library. We first extracted the set of all reads from the raw sequencing output that were associated with the FREQ-Seq^2^ library sequences. This broader set of FREQ-Seq^2^-associated reads was defined as any read pair that contained two individually recognizable barcodes, regardless of whether or not the particular combination was valid, as well as adapter and flanking sequences which each matched the respective reference sequence within an edit distance of four. The rate of usable reads was calculated as the proportion of reads which contained a valid barcode combination and allele and passed the quality control thresholds for matching adapter and flanking sequences, out of the total number of reads derived from the FREQ-Seq^2^ library. After filtering and demultiplexing the reads as described in the Methods, the proportion of useful reads in our control samples was over 91%.

## Discussion

Traditional quantification of allele frequencies by counting colonies is laborious and time-consuming due to the nature of the methods and the sheer number of individual measurements required (Peeler *et al.* 1982; Monsion *et al.* 2008; Jarvis 2016). Our results show that FREQ-Seq^2^ is an effective method for bypassing these problems, while simultaneously improving throughput, repeatability, and cost efficiency. Our method significantly improves upon the scalability of its predecessor, enabling highly multiplexed sample combinations to be analyzed in a single sequencing library, while retaining the original benefits such as simple library preparation and precise quantification.

The barcode redundancy in FREQ-Seq^2^ ensures a high degree of accuracy and minimizes the false positive rate for detecting a given allele. In the hundreds of samples comprising our present results, the great majority of datapoints produced by the method consist of true positives, that is, reads that contain two correct barcodes as well as one of the expected alleles of the target gene. This represents the desired signal, as this indicates that a read corresponds uniquely to one of the barcode combinations with which the library was prepared.

One way to evaluate the efficiency of a method like FREQ-Seq^2^ is to compare the level of each true positive signal to that of the single highest-coverage erroneous group of reads, in which each of the reads’ two individual barcodes are present in the library but are not expected in that particular combination. This provides a useful worst-case noise component as a basis for evaluating the impact of barcoding and sequencing errors on the method because it judges accuracy with respect to the most highly represented class of erroneous reads which actually presents a risk of confounding the analysis of a particular sample (Tanabe and Ishida 2017). The closer the coverage of this error signal is to that of proper reads which uniquely identify a real sample based on a matching barcode pair, the less confidence one has that a particular sample has been accurately measured. Our data demonstrate that FREQ-Seq^2^ performs exceptionally well in this respect.

In evaluating the resolution of FREQ-Seq^2^, it is also useful to note that this noise component is in fact a conservative estimate of the overall error in the dataset. This is due to the fact that many samples exhibit a far lower degree of error than the worst-case, which is based on the coverage for the highest observed erroneous sample that poses a Type I or Type II error risk to any one of our 96 true samples. Indeed, most of the barcode pairs in our samples do not have any barcode in common with this group of spurious reads.

This particular metric does not have any overlap with the various types of obviously erroneous reads, for example, reads that do not contain two individually valid barcodes and aberrant reads due to sequencing, PCR, or ligation error or some other library preparation issue. For these more forgiving classes of errors, the redundancy inherent in FREQ-Seq^2^ allows for unambiguous identification and filtering of erroneous reads. The data show that these error components, despite collectively comprising the most diverse class of non-useful reads, are by and large so low in frequency as to be negligible compared to true positives (Fig. 5a). Additionally, they can be clearly identified and distinguished from a valid FREQ-Seq^2^ read (i.e. reads with a barcode pair corresponding to a known combination in the library), so they can be easily and reliably filtered out from a dataset.

Since the FREQ-Seq^2^ adapter library enables 48^2^ distinct barcode combinations, one can run a very large number of combinations on a single lane of a modern sequencer, providing the latitude and throughput to discard noisier read groups if desired without being constrained by the number of unique identifiers that can be assigned to different samples. Alternatively, replicates of the same libraries can be differentially barcoded to increase and balance sample sizes. In applications where high sensitivity is required, natural random variation in the coverage among samples labeled with particular barcode combinations can be mitigated using such strategies (Matveeva *et al.* 2016; Simonsen *et al.* 2018), as the variation in sample size for different barcode combinations within a given run of the sequencer is in principle random. This is a particularly important characteristic in an Illumina-based method, as undetected barcoding and amplification biases can confound inferences based on coverage and degrade library performance and consistency (Alon *et al.* 2011; Dabney and Meyer 2012).

Indeed, the large sample sizes obtained from this method are another major advantage, one which will only increase with improvements in the read counts and base-pair accuracy of sequencing technologies. Because FREQ-Seq^2^ libraries are prepared such that every read ideally contains two independent barcodes that uniquely identify a sample, in addition to known adapter sequences and an allele at the target locus, every read from the raw output of a sequencer is a potentially usable sample. The efficiency of the method is limited only by the precision of the library preparation and sequencing process itself. In real data, some reads must be discarded due to errors and noise, such as in cases where one or more barcodes do not match or where no target allele is present, and here the large sample sizes combined with the barcode redundancy of FREQ-Seq^2^ are advantageous.

Out of the 96 barcode combinations in our control samples, the most efficient sample had an effective sample size of over 29,000, which was produced from a small fraction of a single lane on a run-of-the-mill short-read sequencer. Additionally, the lowest-coverage sample still had a sample size in the thousands. This indicates that one could further scale the library to contain many more samples than the 96 we included and achieve a larger sample size for each combination than would be possible using traditional quantification methods, without an increase in cost or sequencing resource usage (Wilkening *et al.* 2005; Woods *et al.* 2006; Wasson 2007). At the current levels of sequencing throughput and cost, outstanding quantities of high-precision measurements can be achieved for relatively modest sums (Park and Kim 2016).

Our results show that, compared to a manual approach to estimating allele frequencies by counting colonies, FREQ-Seq^2^ produces much more stable trajectories, while successfully reproducing a qualitatively similar trend consistent with both theory and empirical data for clonal populations evolving towards a fitness peak (Gordo and Campos 2012). The fitness trajectories are likewise qualitatively similar, and we observe similar final values between the two methods. In our evolution experiments, the FREQ-Seq^2^ data exhibits a markedly smoother trajectory for both frequency and fitness across several time points over 2,000 generations. Combined with the small magnitude and uncorrelated nature of its error, FREQ-Seq^2^ provides a substantial reduction in error and increase in precision compared to manually counting colonies. This is not surprising, as the method eliminates unpredictable sources of human and experimental error (Jarvis 2016) while at the same time massively boosting sample sizes.

The allele frequencies at particular loci of interest in a given population can have major effects on the accuracy and outcome of biological inferences, which can go undiscovered if the frequencies are not precisely quantified. For example, it has been shown that the minor allele frequency of a candidate SNP in a genome-wide association study can have a large impact on the likelihood of obtaining a false positive result (Tabangin *et al.* 2009). Additionally, inaccuracies in the determination of allele frequencies in a sample can substantially confound the results and analysis of studies into gene regulatory architecture, population and evolutionary genetic inference, *cis/trans*-variation, and allele-specific expression, among other major topics of active research (Sanjak *et al.* 2017; Steige *et al.* 2017; Zhang and Emerson 2019). Our error analysis illustrates how numerous false positives and false negatives can go undetected without adequate redundancy and sample size, often at rates surpassing common thresholds for statistical significance in large datasets (Fadista *et al.* 2016).

FREQ-Seq^2^ represents a versatile tool for supplementing and validating results and inferences in applications such as high-throughput genetic experiments, long-term evolution studies, genome-wide association studies, allele-specific expression studies, as well as other applications across population, evolutionary, and quantitative genetics.

## Supplementary Material

jkad162_Supplementary_Data

## Data Availability

The FREQ-Seq^2^ plasmid library consisting of two sets of 48 plasmids containing the barcoded adapter fragments is available from Addgene (https://www.addgene.org/browse/article/22444/). The software for demultiplexing paired-end sequencing reads of a FREQ-Seq^2^ library, *fsdm*, is available on GitHub (https://github.com/rnzhao/fsdm). Sequence data from the experiments in this study has been deposited to the NCBI Sequence Read Archive under BioProject accession number PRJNA760234. The FREQ-Seq barcode sequences and the barcode combinations and primers used for the experiments are displayed in Supplementary Tables 1, 2, and Fig. 4. Supplemental material is available at G3 online.
