# Supplementary material for: FREQ-Seq2: a method for precise high-throughput combinatorial quantification of allele frequencies
Source: G3 (Bethesda). 2023 Jul 26;13(10):jkad162. doi: 10.1093/g3journal/jkad162 (PMC10542570; doi:10.1093/g3journal/jkad162)
Supplement: jkad162_Supplementary_Data [file jkad162_supplementary_data.pdf]

Supplementary Material

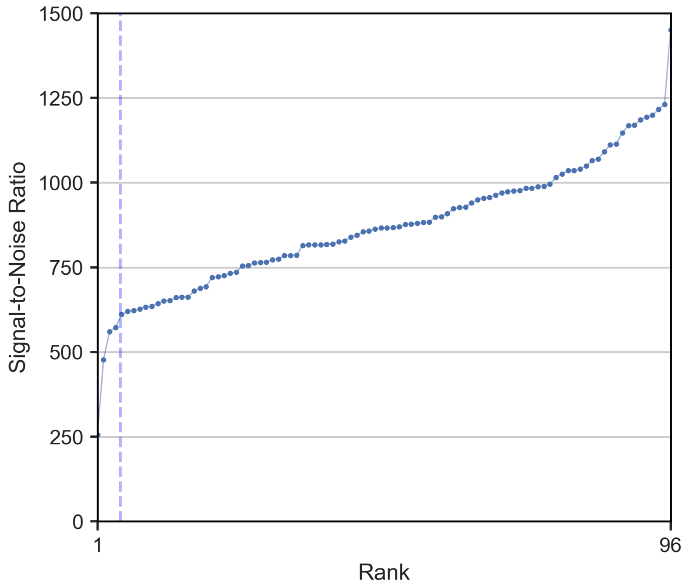

Figure S1. Rank-frequency distribution of signal-to-noise ratio (SNR) for FREQ-Seq^2 read coverage across 96 independent control sample barcode combinations. The dashed blue line indicates the point of the 0.05 quantile.

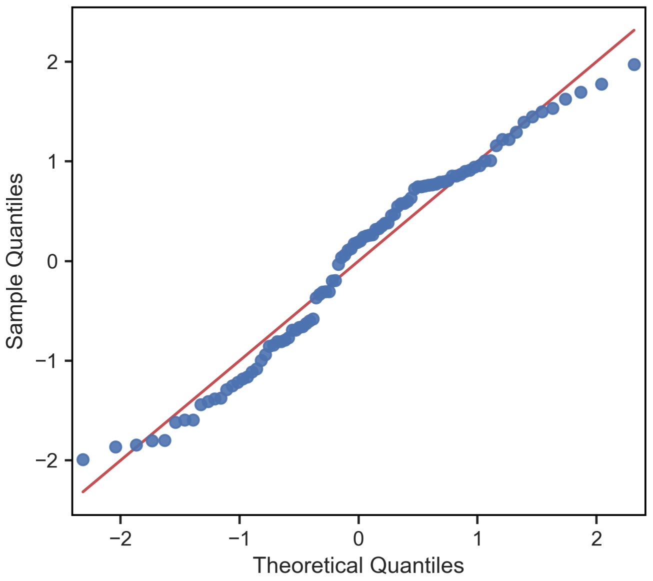

Figure S2. Normal Q-Q plot for error in allele frequency measurements in the control samples.

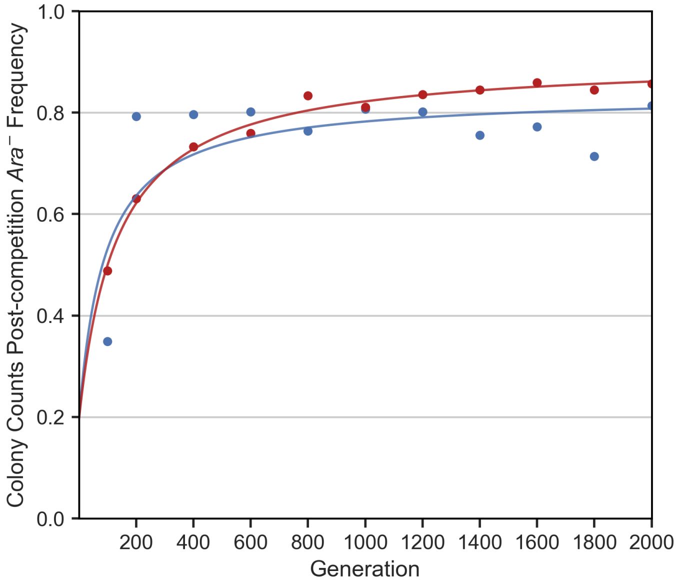

Figure S3. Expected and observed post-competition Ara⁻ allele frequencies for the colony count samples. The blue points are the empirical frequencies measured by colony counting and the red points are the frequencies expected given the measured initial conditions under the independently derived power law model from the FREQ-Seq^2 samples. The curves are the corresponding hyperbolic model fits.

Table S1. Sequences of the 48 FREQ-Seq barcodes.

Label	Sequence
01	AGCAAT
02	CCTGTT
03	GGGTTT
04	GAAGGC
05	ATCTCA
06	ATGGAT
07	ATGTCT
08	CGTGAC
09	TTAGGT
10	GTGCAT
11	AACTTT
12	GGATCG
13	ATAAGG
14	ATTGGT
15	AGTGAG
16	CCCACC
17	CGATGC
18	GATAGC
19	GTCAGA
20	TTAAGC
21	AACCTG
22	CTTTGC
23	TGGAGA
24	AATTGT
25	TGACGA
26	CAAATA
27	GTTCAG
28	CTTCAA
29	GTTGGG
30	GCTTAG
31	TAGCCA
32	TAACTT
33	CGGATA
34	CAGCAG
35	AAGTAG
36	GGGACG
37	CCGTGG
38	ATTGTA
39	TTTAGA
40	CCACGA
41	TCATGG
42	GAACCA
43	TCCTAA
44	CAACGC
45	AGTGTT
46	GGATTA
47	TATATA
48	GTACAA

Table S2. Sequences of the primers, adapters, flanking regions, and alleles for the FREQ-Seq^2 libraries.

Type	Sequence
Barcode 1 amplification primer ('ABC1')	AATGATACGGCGACCAC
Barcode 1 amplification primer ('ABC2')	ACTGGCCGTCGTTTTAC
Barcode 2 amplification primer	AAGCAGAAGACGGCATACG
Barcode 2 amplification primer	GTAAGCAGTGGGTTCTCTAG
Forward adapter sequence	GTAAAACGACGGCCAGT
Reverse adapter sequence	CTAGAGAACCCACTGCTTAC
Upstream allele flanking region	CGGCCAAAATGTGGATCAACG
Downstream allele flanking region	CCTGACCATG
Ara$^-$ / *Ara*$^+$ allele	A / G
